# Non-*Candida* Fungal Prosthetic Joint Infections

**DOI:** 10.3390/diagnostics11081410

**Published:** 2021-08-04

**Authors:** Christos Koutserimpas, Ifigeneia Chamakioti, Stylianos Zervakis, Konstantinos Raptis, Kalliopi Alpantaki, Diamantis P. Kofteridis, Georgia Vrioni, George Samonis

**Affiliations:** 1 Department of Orthopaedics and Traumatology, “251” Hellenic Air Force General Hospital of Athens, 115 25 Athens, Greece; chrisku91@hotmail.com (C.K.); kraptis1981@hotmail.gr (K.R.); 2 Emergency Department, “251” Hellenic Air Force General Hospital of Athens, 115 25 Athens, Greece; ifigenia92@hotmail.com; 3 Department of Cardiology, University Hospital of Heraklion, 714 09 Heraklion, Greece; zervakis_st@hotmail.com; 4 Department of Orthopaedics and Traumatology, “Venizeleion” General Hospital of Heraklion, 714 09 Heraklion, Greece; apopaki@yahoo.gr; 5 Department of Internal Medicine, University Hospital of Heraklion, 715 00 Heraklion, Greece; kofteridisd@hotmail.com; 6 Department of Microbiology, Medical School, National and Kapodistrian University of Athens, 115 27 Athens, Greece; gvrioni@med.uoa.gr

**Keywords:** fungal infection, prosthetic joint infection, *Aspergillus* spp., *Coccidioides* spp., *Pichia* spp., arthroplasty

## Abstract

Background: Fungal prosthetic joint infections (PJIs) are rare, especially those caused by non-*Candida* species. Treatment has not been fully elucidated, since a plethora of antifungal and surgical interventions have been proposed. Τhis study represents an effort to clarify the optimal management of non-*Candida* fungal PJIs, by reviewing all relevant published cases. Methods: A thorough review of all existing non-*Candida* fungal PJIs in the literature was conducted. Data regarding demographics, responsible organisms, antifungal treatment (AFT), surgical intervention, time between initial arthroplasty and onset of symptoms, and time between onset of symptoms and firm diagnosis, as well as the infection’s outcome, were evaluated. Results: Forty-two PJIs, in patients with mean age of 66.2 years, were found and reviewed. *Aspergillus* spp. were isolated in most cases (10; 23.8%), followed by *Coccidioides* spp. (7; 16.7%) and *Pichia* *anomala* (5; 11.9%). Fluconazole was the preferred antifungal regimen (20 cases; 47.6%), followed by amphotericin B (18 cases; 42.9%), while the mean AFT duration was 9.4 months (SD = 7.06). Two-stage revision arthroplasty (TSRA) was performed in 22 cases (52.4%), with the mean time between stages being 5.2 months (SD = 2.9). The mean time between initial joint implantation and onset of symptoms was 42.1 months (SD = 50.7), while the mean time between onset of symptoms and diagnosis was 5.8 months (SD = 14.3). Conclusions: Non-*Candida* fungal PJIs pose a clinical challenge, demanding a multidisciplinary approach. The present review has shown that combination of TSRA separated by a 3–6-month interval and prolonged AFT has been the standard of care in the studied cases.

## 1. Introduction

Joint arthroplasty represents a life-enhancing procedure, providing pain relief and restoration of function, and thereby improving patients’ quality of life. Hip and knee arthroplasty rates are projected to reach 572,000 and 3.48 million, respectively, in the USA by 2030 [1]. Joint reconstruction surgery has evolved over time, encompassing minimally invasive surgical approaches, perioperative pain management and blood transfusion reduction protocols, and navigation or robotic systems, as well as new prosthetic materials [2,3,4]. Nevertheless, complications have not yet been eliminated. Prosthetic joint infections (PJIs) have serious implications on the patient’s quality of life, and in some cases may prove fatal [5].

Fungal organisms are responsible for ~1–2% of PJIs—even less in cases where non-*Candida* species are the cause [6]. Due to the rarity of these infections, no clear guidelines exist regarding management [7]. Currently, on the basis of limited data, a two-stage revision arthroplasty (TSRA) combined with prolonged antifungal treatment (AFT) is suggested [7,8].

The present study represents an effort, by reviewing all published cases of non-*Candida* fungal PJIs, to clarify the medical and surgical treatment options and their success—namely, the eradication of the infection, as well as the maintenance of the viability and functionality of the prosthetic joint, offering the patient the best possible quality of life. The present review takes into account the fact that it covers vast and epidemiologically diverse geographical areas, and that over the course of the long time period studied, medical therapeutic management has changed dramatically.

## 2. Materials and Methods

A meticulous electronic search of the PubMed and MEDLINE databases was conducted to identify all existing articles related to the treatment of non-*Candida* PJIs through to March 2021. Alone and/or in combination, the terms “fungal joint infection”, “fungal prosthetic joint infection”, “fungal knee arthroplasty infection”, “fungal knee infection”, “fungal shoulder arthroplasty infection”, “fungal shoulder infection”, “fungal elbow arthroplasty infection”, “fungal elbow infection”, “fungal hip arthroplasty infection”, and “fungal hip infection” were searched. In addition, terms including each fungal species (e.g., “*Aspergillus* joint infection”, “*Coccidioides* joint infection”, etc.) were also searched.

The review was limited to papers published in English and in peer-reviewed journals. The data extracted from these studies included age, gender, affected joint, responsible non-*Candida* fungal organisms, duration and type of AFT, type of surgical intervention, use of antifungal-agent-loaded cement, time between initial arthroplasty and onset of symptoms, and definitive diagnosis (microscopy/culture/histopathology). Furthermore, the results of medical and surgical treatment, along with the follow-up of each case, were studied and evaluated.

In order to reach a conclusion of the success rates of each surgical treatment, all interventions that failed in each report were thoroughly evaluated. Cases not including surgical interventions were excluded from the assessment of the surgical success rate.

Treatment was considered successful if all signs and symptoms of the infection disappeared and no recurrence was observed during the follow-up period.

Finally, the patients’ Charlson Comorbidity Index was calculated based on the information provided by each study.

Data were recorded and analyzed using Microsoft Excel 2019 (Microsoft Corporation, Redmond, WA, USA).

## 3. Results

A total of 42 cases (18; 43% males), covering a 37-year period (1981–2018), were identified [9,10,11,12,13,14,15,16,17,18,19,20,21,22,23,24,25,26,27,28,29,30,31,32,33,34]. The studied population’s mean age was 66.2 years (standard deviation (SD) = 13.9). The infected joint was the knee in 29 cases (69%), the hip in 12 (29%), and the elbow in 1 (2%). In one case, the knee prosthetic joint infection was bilateral.

Patients’ mean Charlson Comorbidity Index was 3.2 (SD = 1.6).

Furthermore, 26 cases (62%) referred to primary joint reconstruction, while the remaining 16 (38%) were revisions, with the mean number of revisions being 1.5 (SD = 0.7). A total of 10 patients (23.8%) were immunocompromised according to the available information from each report. The mean C-reactive protein (CRP) and erythrocyte sedimentation rate (ESR) at initial presentation were 58.8 mg/L (SD = 68.9) and 57 mm/h (SD = 32.4), respectively. The mean time interval between the initial joint implantation surgery and the symptomatology of the onset of the infection was 42.1 months (SD = 50.7), while the mean time interval between onset of symptoms and firm diagnosis was 5.8 months (SD = 14.3). The mean follow-up was 44.2 months (SD = 24.5).

Regarding the causative fungal organisms, the most frequently isolated were *Aspergillus* spp. in 10 cases (23.8%), followed by *Coccidioides* spp. in 7 (16.7%) and *Pichia anomala* in 5 (11.9%), while *Acremonium* spp., *Alternaria* spp., *Cryptococcus neoformans*, *Histoplasma capsulatum*, *Pseudallescheria* spp., and *Rhodotorula* spp. were each represented by 2 cases (4.8%), and *Aureobasidium* spp., *Malassezia* spp., *Phialemonium curvatum*, *Pithomyces* spp., *Sporothrix schenchii*, *Syncephalastrum racemosum*, and *Trichosporon asahii* were represented by 1 case each (2.4%). Furthermore, one case of unidentified mold was also reported (Table 1). In 12 cases (28.6%), co-infection with bacteria was identified.

Regarding surgical intervention, a two-stage revision arthroplasty (TSRA) was performed in the majority of the reported patients (22 cases (52.4%)), followed by “no surgical intervention” (8 cases (19%)), resection arthroplasty (RA) and debridement (3 cases each (7.1%)), and one-stage revision arthroplasty (OSRA) and arthrodesis (2 cases each (4.8%)) (Table 2). Furthermore, one case (2.4%) of amputation was reported, while in one case (2.4%) the type of surgical intervention was not reported (case 39 from Table 2).

The total success rate of any surgical intervention was 61%. TSRA showed a 66.7% success rate; ORSA, arthrodesis, and debridement had success rates of 66.7% each; and RA had a 50% success rate. In the amputation case, the infection was eradicated. All eight cases (100%) that did not receive surgical intervention were considered, by the authors, as successfully treated only by medical means. It is of note that all of these eight patients were put on lifelong antifungal suppressive treatment (Table 2).

More specifically regarding TSRA, the mean time interval between the initial removal of the implants and the final re-implantation was 5.2 months (SD = 2.9). Regarding the cement spacer used, in nine cases an antifungal regimen was impregnated in the cement; amphotericin B was used in five of these cases, voriconazole in three, and itraconazole in one.

Regarding antifungal treatment (AFT), in 20 cases (47.6%), a single agent was used; in 15 (35.7%), two—either simultaneously or consecutively—while in 1 (2.4%), more than two agents were used. In six cases the information regarding the specific antifungal drug was not available (cases 3, 4, 14, 15, 24, and 35 in Table 2). The mean duration of AFT was 9.4 months (SD = 7.06), while it is of note that in eight cases a lifelong suppressive AFT was started (cases 17, 18, 19, 21, 22, 24, 25, and 27 in Table 2).

Fluconazole was the preferred agent in 20 cases ((47.6%), in 8 (40%) as monotherapy), followed by amphotericin B in 18 cases ((42.9%), in 3 (16.7%) as monotherapy), voriconazole in 8 ((19%), in 2 (25%) as monotherapy), and itraconazole in 5 ((11.9%), in 3 (60%) as monotherapy). Caspofungin and posaconazole, as monotherapy, were preferred in one case (2.4%) each. The final outcome was successful in 33 cases (78.6%). Regarding the most prevalent fungus, treatment was successful in 90% of *Aspergillus* spp., and in 100% of *Coccidioides* spp. and *Pichia anomala*.

The details of AFT are exhibited in Table 2. In the most frequently isolated *Aspergillus* spp. cases, the preferred AFT was fluconazole (5 cases (50%), in 3 (66.7%) as monotherapy), followed by amphotericin B (4 cases (40%), in 2 (50%) as monotherapy), and posaconazole, voriconazole, and caspofungin, which were given all as monotherapy in one case (10%) each. The majority of patients with *Coccidioides* spp. Infections received fluconazole (4 cases (57%), in 3 (75%) as monotherapy), followed by amphotericin B (2 cases (28.6%), in 1 (50%) as monotherapy) and itraconazole (1 case (14.3%) as monotherapy). Most patients with *Pichia anomala* were treated with fluconazole (5 cases (100%), in 1 (20%) as monotherapy), followed by amphotericin B (4 cases (80%), none as monotherapy).

## 4. Discussion

Fungal PJIs are uncommon and extremely challenging regarding their management [5,6]. Most such infections are caused by *Candida* species [5,35]. Hence, limited data and information exist regarding PJIs caused by non-*Candida* organisms [8]. The present study aims, by reviewing published data, to clarify the characteristics, treatment options, and outcomes of non-*Candida* fungal PJIs.

PJIs, along with other invasive fungal infections, represent a major cause of morbidity and mortality in current medical practice. Optimal treatment of fungal PJIs remains unclear, since no certain guidelines exist regarding the antifungal regimen and the indicated surgical intervention [7,8]. TSRA and long-term AFT are proposed due to lack of data [5,7,8]. Information about the kind of AFT, its duration, and its success rate, as well as type of surgical intervention, the use of antifungal agents in cement, and the time intervals between the two stages of TSRA, are of utmost importance for the clarification of the best medical treatment and the improvement of the surgical management of these cases.

The present study has reviewed 42 cases of non-*Candida* fungal PJIs over 37 years, with a mean follow-up of 44.2 months. The incidence of fungal PJIs incidence is expected to rise, due to the increasing number of prosthetic joint reconstruction surgeries worldwide [1,8]. Immunosuppression and systemic disease have been widely acknowledged as risk factors for invasive fungal infections, while revision joint surgeries increase the risk of infection [5]. In the present study, a total of 10 patients (23.8%) were immunocompromised, while in 38% of the cases a revision reconstruction surgery had been performed.

Fungal PJI is frequently of hematogenous origin [8,14,16]. However, intraoperative contamination by fungal skin pathogens may also occur [36]. If the contamination originates in the skin, symptoms usually appear early in the postoperative period. In the studied cases, the mean time interval between initial joint reconstruction surgery and onset of symptoms was 42.1 months (ranging from 0.7 to 120), supporting the theory of hematogenous spread. The mean time from onset of symptoms to firm diagnosis was 5.8 months. Fungal PJIs usually present with indolent symptoms and, therefore, diagnosis may be delayed.

In the present review, the predominant fungi involved were *Aspergillus* spp., identified in 10 cases (23.8%), followed by *Coccidioides* spp. in 7 cases (16.7%) and *Pichia anomala* in 5 (11.9%). In 12 cases (28.6%), co-infection with bacteria was diagnosed. Concomitant bacterial infection has already been reported in 15–20% of fungal PJI cases [37]. *Coccidioides* spp. are geographically restricted dimorphic fungi represented rather heavily in the present review. However, it must be noted that most of the published cases originate in the United States, where these fungi are endemic.

Invasive *Aspergillus* infections are typically seen in patients with significant underlying immunosuppression [13,14,17]. However, in the present review, most of the reported *Aspergillus* PJIs occurred in immunocompetent individuals.

Several surgical intervention options have been described for the treatment of fungal PJIs [7,8]. In the reviewed population, TSRA was preferred in most cases (52.4%), with a success rate of 66.7%. The mean time between the two surgical stages was 5.2 months, and in nine cases cement impregnated with an antifungal agent was used. ORSA, arthrodesis, and debridement each exhibited a 66.7% success rate, while for RA it was 50%. Eradication of the infection was the result in one case of amputation.

It is of note that arthrodesis, RA, and amputation, although proven to be successful treatment options—since the infection was finally eradicated—are associated with a high negative impact on the patient’s quality of life, and may possibly lead to loss of independence. Furthermore, in the eight cases that did not receive any surgical intervention (cases 15, 17, 19, 21, 22, 24, 27, and 40, shown in Table 1 and Table 2), although the treatment’s outcome was considered by authors to be successful, all patients subsequently commenced lifelong fungal suppression; therefore, it is understood that definitive eradication of the infection was not achieved. These reports raise the question of whether lifelong fungal suppression by AFT is a feasible option in cases where further surgery may lead to failure, either due to technical difficulties (e.g., extensive bone loss, challenging reconstructive options) or to patient comorbidities.

Guidelines for the treatment of fungal osteoarticular infections exist; however, no clear recommendations are available for the treatment of such PJIs [7]. Therefore, the duration of treatment is mainly based on the clinical and laboratory findings of each case and the physicians’ experience with such infections. It is, therefore, of utmost importance to carry out susceptibility testing to obtain accurate MIC values following the isolation of the fungus, taking into account that different species of fungi (e.g., yeast, molds, etc.) are characterized by intrinsic resistance to certain antifungal compounds [8]. Additionally, it must be noted that for a number of molds, laboratory methods indicating MICs are not standardized and unanimously accepted, while the immune status of the patient plays a major role [38].

In all of the reviewed cases, the causative fungus was identified. However, the microbiological procedures used were not described in most of them. Moreover, since the present review spans almost four decades, it is understandable that identification techniques have evolved over time, ranging from simple microscopy, pathology, and culture to modern molecular methods [38]. Hence, although the information about the fungal cause of cases is sufficient, information about specific identification procedures has been inadequate.

Regarding AFT, fluconazole was the preferred antifungal agent (47.6%), followed by amphotericin B (42.9%). Fluconazole was extensively used in the reviewed cases, although this agent is ineffective against molds. However, it must be taken into account that fluconazole and amphotericin B deoxycholate were the only available agents in the early years of the reviewed cases. Fluconazole has on rare occasions been associated with severe hepatotoxicity [39]. Hence, liver function tests should be performed regularly during prolonged fluconazole therapy, while amphotericin B, although an effective broad spectrum regimen, is relatively toxic, and its side effects—including renal dysfunction—may restrict its long-term use, which is essential for PJI cases [40]. The liposomal compounds of amphotericin B have reduced the drug’s nephrotoxicity considerably, but long-term use of these agents may be still problematic [40]. Voriconazole, which was introduced in 2003, has proven to be the drug of choice against *Aspergillus* spp. This agent has changed the management of *Aspergillus* infections dramatically over the past several decades; with all the characteristics of azole compounds, it is moderately hepatotoxic and much less nephrotoxic than all amphotericin compounds [41].

The mean AFT duration was found to be 9.4 months. The final outcome was successful in 33 cases (78.6%). It is of note, however, that the success rate drops to 66.7% in cases of bacterial co-infection. Concomitant bacterial infections occur in between 15 and 20% of fungal cases, while the poor prognosis for co-infective PJIs has already been underlined [42].

The present review has some limitations. There is heterogeneity between different joint reconstruction surgeries (hip, knee, and elbow), while not all information was available from each case, such as specific signs and symptoms of the infection, antifungal dosages, mode of administration, monitoring of serum levels, MICs, and adverse events. Another point not clarified is the specific microbiological techniques used for fungal identification (especially modern ones, such as PCR and/or β-D-glucan). Nevertheless, this study reviews all of the non-*Candida* fungal PJIs in a systematic way, offering valuable insights regarding epidemiology, severity, surgical management, and medical treatments that changed dramatically over the long study period, as well as outcomes.

It is important that over the past several decades a number of new antifungal agents (e.g., voriconazole, posaconazole, echinocandins, and isavuconazole) were introduced, providing more medical treatment options, hopefully associated with better results [41].

The present review shows that non-*Candida* fungal PJIs represent a very challenging clinical entity. A combination of the proper medical AFT, based on susceptibility testing (when feasible) and surgical intervention, seems to represent the current standard management. There have been reports of successful treatment of such cases with OSRA and debridement. However, TSRA should be strongly recommended. The combination of TSRA separated by 3–6 months and a prolonged period of AFT is suggested on the basis of limited data. Lifelong fungal suppression with a proper agent (based on the type of the fungal species) is suggested in cases where surgery is not desirable (either due to patient comorbidities or technical difficulties predicting uncertain results). More data and research are needed, focusing on proper treatment—since the results of therapeutic procedures and policies, such as the antifungal-loaded cement spacers and AFT duration, remain unclear—in order to conclude the optimal management approach.

## Figures and Tables

**Table 1:** Patients’ demographics, comorbidities, responsible fungus, affected joint, bacterial co-infection, time (T) intervals from joint implantation to symptom onset and from symptom to diagnosis, number of previous revisions in the same joint, C-reactive protein, (CRP), and erythrocyte sedimentation rate (ESR) at presentation.

Case No	Year	Author	Country of Origin	Gender/Age	Fungus	Joint	Co-Infection	CRP mg/L	ESR mm/h	Charlson Comorbidity Index	Immunosuppressive Medication and Conditions	Number of Previous Revisions	T from Implantation to Symptomatology (Months)	T from Symptom Onset to Diagnosis (Months)
1	2014	Cheng-Yi Wu et al. [9]	Taiwan	M/47	*Acremonium* spp.	Hip	*Penicillium* species	16.17	127	3	Liver cirrhosis and chronic HBV infection	-	120	2
2	2018	Zhisen Gao et al. [10]	China	F/52	*Acremonium strictum*	Knee	-	0.348	17	1	-	1	-	-
3	2018	Brown et al. [11]	USA	M/54	*Alternaria* spp.	Knee	-	35	36	1	-	-	-	-
4	2018	Brown et al. [11]	USA	F/55	*Alternaria* spp.	Knee	-	35	36	1	-	Yes (NA)	-	-
5	2016	Geng et al. [12]	China	F/ 63	*Aspergillus* spp.	Knee	-	49.9	25	2	-	-	-	-
6	2018	Zhisen Gao et al. [10]	China	F/63	*Aspergillus* spp.	Knee	-	4.99	25	2	Diabetes Mellitus	3	-	-
7	2018	Zhisen Gao et al. [10]	China	M/63	*Aspergillus* spp.	Knee	Gram-positive bacteria, *mycobacterium*	10	92	4	-	2	-	-
8	1992	Austin et al. [13]	USA	M/80	*Aspergillus fumigatus*	Knee	-	-	100	4	Megaloblastic anemia, neutropenia, immunosuppressive therapy for the past five years	-	25	1
9	2001	Baumann et al. [14]	USA	F/27	*Aspergillus fumigatus*	Knee	-	37	55	2	-	1	53	0.1
10	2011	Yilmaz et al. [15]	Turkey	M/81	*Aspergillus fumigatus*	Knee	-	40	108	4	-	-	12	0.3
11	2012	Hwang et al. [16]	Korea	F/74	*Aspergillus. fumigatus*	Knee	-	63	32	4	-	-		
12	2017	Bartash et al. [17]	USA	Μ/54	*Aspergillus terreus*	Hip	*Streptococcus mitis*	24	56	3	-	1	10	3
13	2017	Kwong et al. [18]	Canada	F/64	*Aspergillus terreus*	Elbow	Coagulase-negative *staphylococcus* (CONS)	8.7	29	3	Prednisone	1	192	6
14	2018	Brown et al. [11]	USA	F/88	*Aspergillus versicolor*	Knee	*S. lugdunensis*	35	36	4	-	-	-	-
15	2018	Brown et al. [11]	USA	M/60	*Aureobasidium* spp.	Hip	*Hormonema*	218	52	2	-	-	-	-
16	2009	B.Johannsson & J. Callaghan [19]	USA	M/84	*Cryptococcus neoformans*	Hip	-	50.8	43	8	-	1	108	0.5
17	2014	Shah et al. [20]	USA	F/77	*Cryptococcus neoformans*	Hip	-	-	-	5	Azathioprine and prednisone	1	3	0.3
18	2011	Kuberski et al. [21]	USA	F/84	*Coccidioides* spp.	Knee	-	-	-	4	-	-	24	-
19	2011	Kuberski et al. [21]	USA	M/72	*Coccidioides* spp.	Knee	-	-	-	5	Rheumatoid arthritis on chronic prednisone	-	60	0.3
20	2011	Kuberski et al. [21]	USA	M/66	*Coccidioides* spp.	Hip	-	-	-	2	-	2	12	5
21	2011	Kuberski et al. [21]	USA	M/37	*Coccidioides* spp.	Knee	-	-	-	1	-	-	24	-
22	2013	Austen et al. [22]	The Netherlands	F/77	*Coccidioides immitis*	Knee	-	4	32	3	-	-	71	5
23	2015	Arbeloa-Gutierrez et al. [23]	Spain	M/74	*Coccidioides* spp.	Knee	-	-	132	3	Adrenal insufficiency, corticosteroids	-	84	-
24	2018	Brown et al. [11]	USA	M/89	*Coccidioides immitis*	Hip	-	218	52	4	-	-	-	-
25	1998	Fowler et al. [24]	USA	F/84	*Histoplasma capsulatum*	Hip	-	-	51	5	Prednisone-dependent polymyalgia rheumatica, granulomatous disease	2	96	60
26	2017	Nowbakht et al. [25]	USA	M/77	*Histoplasma capsulatum*	Knee	Group B *Streptococci*	-	-	5	-	3	8	0.3
27	2016	Leylabadlo et al. [26]	Iran	F/59	*Malassezia* spp.	Knee	-	2.33	-	1	-	-	0.7	-
28	2016	Geng et al. [12]	China	F/76	Mould (unidentified)	Knee	Coagulase-negative *staphylococcus*	65	80	4	-	-	8	-
29	2012	Anagnostakoset al. [27]	Germany	M/64	*Phialemonium curvatum*	Knee	-	>20	-	2	-	1	-	-
30	2012	Hwang et al. [16]	Korea	F/49	*Pichia anomala*	Knee	-	48	42	1	-	-	-	
31	2015	Q.-J. Wang et al. [28]	China	M/68	*Pichia anomala*	Knee	-	48	38	2	-	-		
32	2018	Hwang et al. [16]	Korea	F/73	*Pichia anomala*	Knee		41	45	4	-	-		
33	2018	Hwang et al. [16]	Korea	F/ 66	*Pichia anomala*	Knee (bilateral)	*C. lusitaniae*	15	18	3	-	-		
34	2018	Hwang et al. [16]	Korea	F/70	*Pichia anomala*	Knee	-	32	93	4	-	-		
35	2018	Brown et al. [11]	USA	F/77	*Pithomyces* spp.	Hip	*Propionobacterium acnes*	218	52	3	-	-	-	-
36	2011	Gottesman-Yekutieli et al. [29]	Israel	F/56	*Pseudallescheria boydii*	Hip	-	169	-	2	Chronic joint disease on prednisone and methotrexate	-	12	12
37	2011	Lackner et al. [30]	Austria	M/61	*Pseudallescheria apiosperma*	Knee	-	200	102	2	-	-	1.2	1
38	2018	Hwang et al. [16]	Korea	F/75	*Rhodotorula minuta*	Knee	MRSA	29	71	4	-	-		
39	2008	Savini et al. [31]	Italy	F/41	*Rhodotorula mucilaginosa*	Hip	-	-	-	6	Human immunodeficiency virus	1		0.5
40	1995	DeHart [32]	USA	M/56	*Sporothrix schenckii*	Knee	-	-	-	1		-	-	
41	2002	Ceffa et al. [33]	Italy	F/72	*Syncephalastrum racemosum*	Knee	*Corynebacterium* group	-	-	4	-	1	2	
42	2015	Zuo et al. [34]	China	F/73	*Trichosporon asahii*	Knee	-	27.2	32	5			1	0.5

**Table 2:** Surgical and antifungal treatment, follow-up, and infection outcome of the reported cases. ST: surgical treatment; TSRA: two-stage revision arthroplasty; OSRA: one-stage revision arthroplasty; AFT: antifungal treatment; LS: lifelong suppression; NS: no surgery; RA: resection arthroplasty; NA: not available.

Case	ST	Time between Stages in TSRA (Months)	Antifungal Regimen in Cement	Antifungal Treatment (AFT)	Total Duration of AFT (Months)	Follow-Up (Months)	Outcome
1	TSRA	4	-	Fluconazole	15	12	Success
2	TSRA	9	Voriconazole	Voriconazole, Fluconazole	6.5	30	Success
3	TSRA	6	Amphotericin B	NA	-	60	-
4	TSRA	6	Amphotericin B	NA	-	60	-
5	TSRA	7	-	Fluconazole	7	62	Success
6	TSRA	7	-	Fluconazole	8.5	80	Success
7	TSRA (2 × spacer exchange before final implantation)	14	-	Fluconazole	3	51	Failure
8	RA	-	-	Amphotericin B	3	-	Success
9	TSRA	3.5	-	Amphotericin B, Fluconazole	10.5	60	Success
10	TSRA	4	-	Amphotericin B	1.5	48	Success
11	TSRA	3	-	Amphotericin B, Fluconazole	-	67	Success
12	TSRA	4	Voriconazole	Posaconzole	> 1	-	-
13	RA (2 × TSRA failed prior to RA)	-	-	Voriconazole, Caspofungin	-		Success
14	TSRA	6	Amphotericin B	NA	-	60	-
15	NS (no surgery)	-	-	AFT Suppression (NA)	-	60	Success
16	RA	-	-	Amphotericin B	-	10	Failure
17	NS	-	-	Amphotericin B, fluconazole	LS	12	Success
18	Arthrodesis	-	-	Amphotericin B	LS	48	Success
19	NS	-	-	Amphotericin B, Fluconazole	LS	48	Success
20	OSRA (×2, failed the first time)	-	-	Fluconazole	17	12	Success
21	NS	-	-	Fluconazole	LS	96	Success
22	NS	-	-	Fluconazole	LS	6	Success
23	Arthrodesis	-	Amphotericin B	Itraconazole	-	6	Success
24	NS	-	-	-	LS	60	Success
25	Debridement	-		Itraconazole	LS	36	Success
26	TSRA	9	Voriconazole	Itraconazole	24	24	Success
27	NS	-	-	Amphotericin B, Fluconazole, Voriconazole	LS	-	Success
28	TSRA	3	-	Fluconazole	2.5	44	Success
29	OSRA	-	-	Voriconazole	6	5	Success
30	TSRA	2.5	-	Amphotericin B, Fluconazole	-	45	Success
31	TSRA	6	Amphotericin B	Fluconazole	3	65	Success
32	TSRA	2.5	-	Amphotericin B, Fluconazole	-	67	Success
33	TSRA	2	-	Amphotericin B, Fluconazole	-	35	Success
34	TSRA	4	-	Amphotericin B, Fluconazole	-	67	Success
35	Debridement	-	-	NA	-	60	Success
36	TSRA	6.5	Itraconazole	Voriconazole	10	24	Success
37	Amputation (1 × RA and 1 × arthrodesis both failed)	-	-	Itraconazole, Voriconazole	5.5	96	Success
38	TSRA	2.5	-	Amphotericin B, Fluconazole	-	26	Failure
39	NA	-	-	Amphotericin B	> 0.75	-	NA
40	NS	-	-	Amphotericin B, Itraconazole	24	30	Success
41	TSRA	2.5	-	Amphotericin B, Voriconazole	-	36	Success
42	Debridement	-	-	Amphotericin B, Voriconazole	12	26	Failure

## Data Availability

Not applicable.
